# Influence of a Short-Term Iron-Deficient Diet on Hepatic Gene Expression Profiles in Rats

**DOI:** 10.1371/journal.pone.0065732

**Published:** 2013-06-05

**Authors:** Asuka Kamei, Yuki Watanabe, Kaori Kondo, Shinji Okada, Fumika Shinozaki, Tomoko Ishijima, Yuji Nakai, Takashi Kondo, Soichi Arai, Keiko Abe

**Affiliations:** 1 Project on Health and Anti-aging, Kanagawa Academy of Science and Technology Life Science & Environment Research Center 4F C-4, Kawasaki, Kanagawa, Japan; 2 Research Center for Allergy and Immunology, Institute of Physical and Chemical Research (RIKEN), Yokohama, Kanagawa, Japan; 3 Graduate School of Agricultural and Life Sciences, The University of Tokyo, Tokyo, Japan; 4 General Research Institute, Tokyo University of Agriculture, Setagaya-ku, Tokyo, Japan; Wageningen University, The Netherlands

## Abstract

Iron is an essential mineral for the body, and iron deficiency generally leads to anemia. However, because non-anemic iron deficiency can exist, we performed a comprehensive transcriptome analysis of the liver to define the effects of this condition on the body. Four-week-old male rats were fed a low-iron diet (approximately 3 ppm iron) for 3 days and compared with those fed a normal diet (48 ppm iron) by pair feeding as a control. The rats in the iron-deficient diet group developed a non-anemic iron-deficient state. DNA microarray analysis revealed that during this short time, this state conferred a variety of effects on nutrient metabolism in the liver. In comparison with long-term (17 days) iron-deficiency data from a previous study, some of the changed genes were found to be common to both short- and long-term iron deficiency models, some were specific to the short-term iron deficiency model, and the others were oppositely regulated between the two feeding terms. Taken together, these data suggest that although the blood hemoglobin level itself remains unchanged during non-anemic iron deficiency, a variety of metabolic processes involved in the maintenance of the energy balance are altered.

## Introduction

Iron is an essential modulator of metabolic and physiological functions by acting as a cofactor for many proteins [1], [2]. Prolonged periods of iron deficiency result in a smaller iron storage pool, the suppression of hemoglobin biosynthesis and the development of anemia. We previously reported that dietary iron deficiency-induced anemia causes a variety of changes in nutrient metabolism and can ultimately lead to apoptosis in the liver, potentially as a result of endoplasmic reticulum (ER) stress [3]. These results indicated that iron is an essential nutrient associated with a variety of biological activities.

Anemia results from prolonged iron deficiency. In Japan, approximately 40% of women suffer from non-anemic iron deficiency, and 10% suffer from iron-deficient anemia [4]. Because hemoglobin levels do not change during non-anemic iron deficiency, this condition is not recognized as a serious problem. However, because iron is essential for the biochemical activation of enzymes [1], [2], such as cytochromes, even non-anemic iron deficiency is expected to cause some physiological effects.

Because the liver is the principal organ in which iron is stored, a variety of metabolic changes are expected to occur in the liver as a result of an iron-deficient diet. A holistic understanding of these changes requires an analytical method that can be use to comprehensively examine specific aspects of the phenomenon. We determined that a DNA microarray analysis was the best technique to examine the global transcriptional changes occurring in an iron-deficient animal. Using this technique, we analyzed the hepatic gene expression profiles of rats fed a short-term iron-deficient diet. Here, we report that even the non-anemic iron deficiency that was induced by this diet caused a variety of changes in nutrient metabolism in the liver. We also compared the results obtained from short- and long-term iron-deficiency conditions [3] to identify similarities and differences in the resulting hepatic gene expression profiles.

## Results

### Blood hemoglobin, serum iron, TIBC and ferritin levels

No significant hemoglobin level changes were observed between the pair-fed control group and the iron-deficient group. The total iron binding capacity (TIBC), serum iron and ferritin levels in the iron-deficient group were 118%, 32% and 74% of those in the pair-fed group, respectively (*P*<0.01 for TIBC, *P*<0.05 for serum iron and ferritin) (Table 1). Therefore, the rats in the iron-deficient diet group exhibited no anemia despite their iron deficiency. The liver iron level in the iron-deficient group was 64% of that of the pair-fed group (*P* = 0.14) (Table 1). No significant changes were observed in the total cholesterol, triacylglycerol or total bile acid levels in the liver or in the total cholesterol, triacylglycerol, glucose or pyruvate levels in the serum (Table 2).

**Table 1:** Body weight, liver weight, liver iron, hemoglobin, TIBC, serum iron and serum ferritin levels.

	Pair-fed group	Iron-deficient group
Body weight, g	105.1±1.2	104.9±2.1
Liver weight, g	3.97±0.13	4.45±0.13*
Liver iron, µg/g wet liver	38.02±7.55	24.16±1.53
Hemoglobin, g/dl	10.69±0.53	10.17±0.31
TIBC, µg/dl	600.0±7.8	708.0±15.3**
Serum iron, µg/dl	153.6±30.6	48.8±4.8*
Serum ferritin, ng/ml	1421.7±65.0	1049.0±154.6*

The values represent the mean ± SEM (n = 5).

* *P*<0.05 and

** *P*<0.01 for between-diet group differences.

**Table 2:** Total cholesterol, triacylglycerol and total bile acid levels in the liver and total cholesterol, triacylglycerol, glucose and pyruvate levels in serum.

		Pair-fed group	Iron-deficient group
Liver	Total cholesterol, mg/g wet tissue	1.52±0.03	1.59±0.12
	Triacylglycerol, mg/g wet tissue	4.95±0.37	4.86±0.66
	Total bile acid, µg/g wet tissue	0.16±0.02	0.14±0.00
Serum	Total cholesterol, mg/dl	74.6±8.0	86.2±2.5
	Triacylglycerol, mg/dl	49.6±14.5	62.8±8.6
	Glucose, mg/dl	150.6±1.2	147.8±9.0
	Pyruvate, mg/dl	2.11±0.22	1.85±0.29

The values represent the mean ± SEM (n = 5).

### DNA microarray data quantification and detection of differentially expressed probe sets

We quantified the raw microarray data (Affymetrix CEL files) using a robust multiarray average (RMA). A hierarchical clustering analysis revealed that the pair-fed group formed two clusters. The iron-deficient group formed two clusters; however, one member of this group was located in a cluster of the pair-fed group (Figure S1). By applying the rank products (RP) method to the RMA quantified data, we selected 91 probe sets that were up-regulated and 186 that were down-regulated (FDR<0.05) in the iron-deficient group relative to the pair-fed group (Table S1).

### Gene ontology analysis

**Figure 1:**
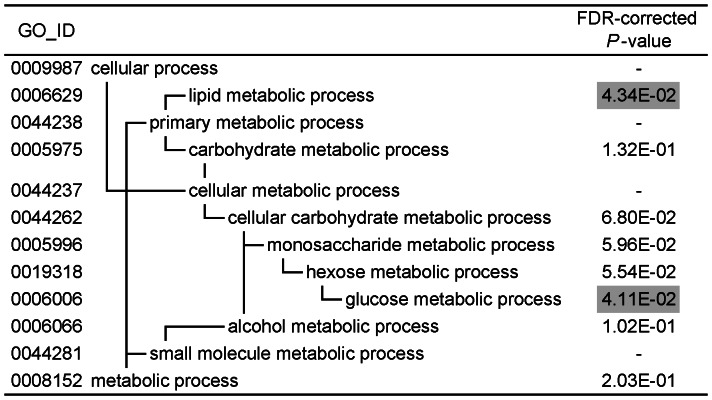
GO terms associated with the genes that were up-regulated in the iron-deficient group. FDR-corrected *P*-values were defined by the modified Fisher's exact test with the Benjamini and Hochberg FDR correction. FDR-corrected *P*-values<0.05 are shaded in gray.

**Figure 2:**
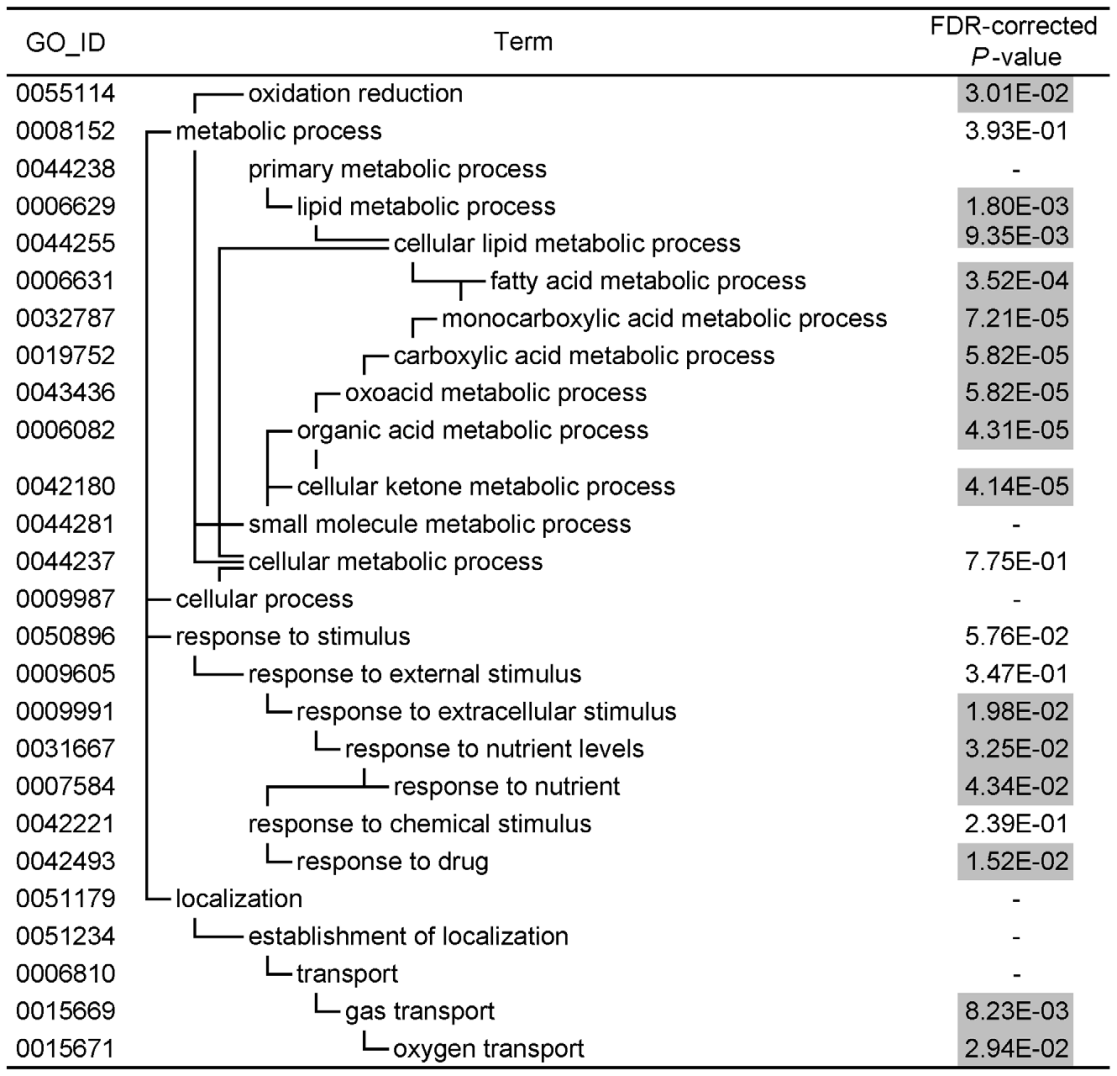
GO terms associated with the genes that were down-regulated in the iron-deficient group. FDR-corrected *P*-values were defined by the modified Fisher's exact test with the Benjamini and Hochberg FDR correction. FDR-corrected *P*-values<0.05 are shaded in gray.

To identify gene ontology (GO) terms that were overrepresented among the differentially expressed genes, we performed a gene-annotation enrichment analysis using the online software program DAVID (the Database for Annotation, Visualization, and Integrated Discovery). The GO terms that were significantly enriched in the genes that were up- and down-regulated in the iron-deficient treatment are summarized in Figures 1 and 2. The hierarchical structure of GO facilitated the identification of more specific GO terms that appeared deeper in the hierarchy. The GO terms that were significantly enriched in the genes up-regulated by the iron-deficient diet were glucose metabolic process (GO: 0006006) and lipid metabolic process (GO: 0006629). The GO terms that were significantly enriched in the genes down-regulated by this diet were oxidation reduction (GO: 0055114), lipid metabolic process (GO: 0006629), organic acid metabolic process (GO: 0006082), cellular ketone metabolic process (GO: 0042180), response to extracellular stimulus (GO: 0009991), response to drug (GO: 0042493) and gas transport (GO: 0015669).

### Gene expression profile details

**Figure 3:**
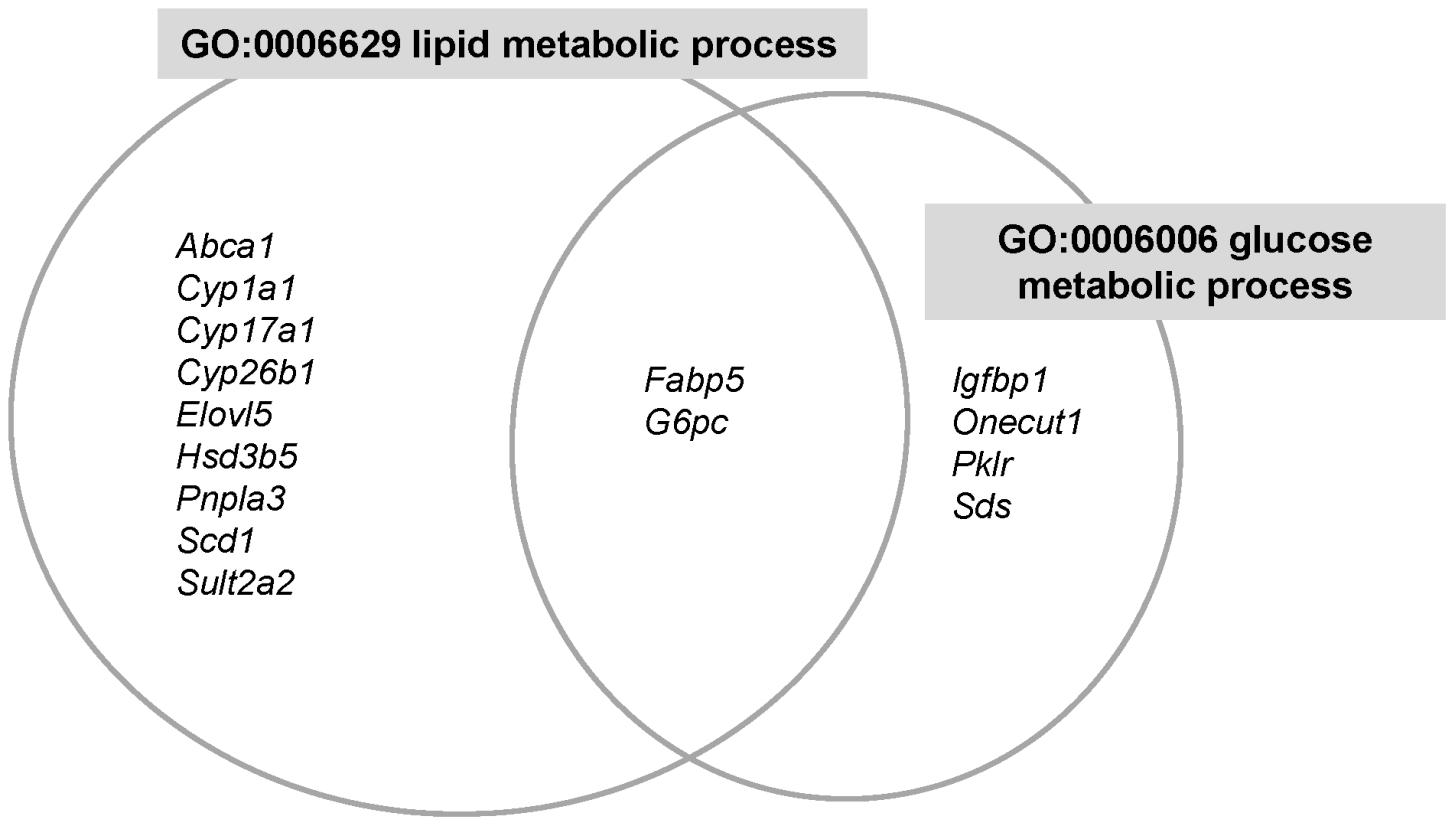
Venn diagrams representing the association of up-regulated genes with multiple GO terms. The resulting complex interdependencies of categories were shared with differentially expressed genes in the case of non-anemic iron deficiency. The genes are represented as gene symbols.

**Figure 4:**
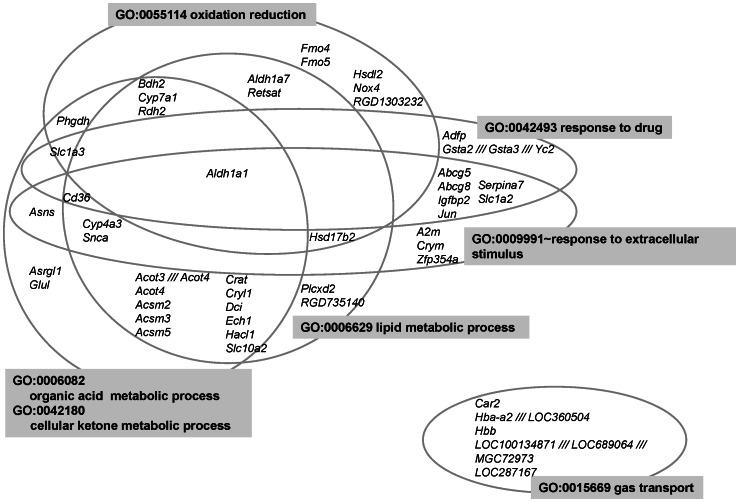
Venn diagrams representing the association of down-regulated genes with multiple GO terms. The resulting complex interdependencies of categories were shared with differentially expressed genes in the case of non-anemic iron deficiency. The genes are represented as gene symbols.

The up- and down-regulated genes, which were categorized according to their functional annotations, are shown in Figures 3 and 4, and further details are provided below.

### Up-regulated genes

Glucose metabolism was one of the GO terms enriched among the up-regulated genes; two of the associated genes were genes encoding glucose-6-phosphatase, catalytic subunit (*G6pc*) and pyruvate kinase, liver and RBC (*Pklr*). These enzymes are rate-limiting in gluconeogenesis [5] and glycolysis [6], respectively. However, *G6pc* up-regulation was not accompanied by a corresponding increase in the serum glucose level (Table 2). The level of serum pyruvate, which is converted from phosphoenolpyruvate by pyruvate kinase and from L-serine by serine dehydratase (*Sds*), was also unchanged (Table 2).

With respect to lipid metabolism, stearoyl-coenzyme A desaturase 1 (*Scd1*) and ELOVL family member 5, elongation of long chain fatty acids (yeast) (*Elovl5*), which are involved in fatty acid desaturation, were up-regulated.

### Down-regulated genes

Genes associated with organic acid metabolism and cellular ketone metabolism, including the fatty acid catabolism genes acyl-CoA synthetase medium-chain family members 2, 3 and 5 (*Acsm2*, *Acsm3* and *Acsm5*, respectively); enoyl-CoA hydratase 1, peroxisomal (*Ech1*); 2-hydroxyacyl-CoA lyase 1 (*Hacl1*); dodecenoyl-CoA delta isomerase (*Dci*); cytochrome P450, family 4, subfamily a, polypeptide 3 (*Cyp4a3*); and carnitine acetyltransferase (*Crat*), were all down-regulated. Acyl-CoA thioesterase 3 and 4 (*Acot3* and *Acot4*), which are involved in fatty acid biosynthesis, were also down-regulated. After 3 days of receiving the experimental diet, the animals' serum and hepatic triacylglycerols remained at normal levels (Table 2). In contrast, in the anemic animals, genes encoding enzymes involved in fatty acid biosynthesis were down-regulated [3].

Several genes related to amino acid metabolism, namely, phosphoglycerate dehydrogenase (*Phgdh*), asparagine synthetase (*Asns*) and glutamate-ammonia ligase (*Glul*), were down-regulated. Solute carrier family 1 (glial high affinity glutamate transporter), members 2 and 3 (*Slc1a2* and *Slc1a3*), which are associated with aspartate and glutamate transport, were also down-regulated.

Aldehyde dehydrogenase 1 family, member A7 (*Aldh1a7*), the enzyme that converts acetaldehyde to acetate in the major oxidative pathway of alcohol metabolism, was down-regulated. Acyl-CoA synthetase converts acetate to acetyl-CoA, which is used in the biosynthesis of cholesterol and fatty acids. Cytochrome P450, family 7, subfamily a, polypeptide 1 (*Cyp7a1*), the rate-limiting enzyme of cholesterol catabolism and thus bile acid biosynthesis [7], was also down-regulated. The expression of the genes encoding LXR, a transcription factor for *Cyp7a1*, and FXR, a repressor of *Cyp7a1* [7], was unchanged significantly. The serum and liver cholesterol and bile acid levels did not change significantly (Table 2).

In terms of the responses to drugs and extracellular stimuli, ATP-binding cassette, sub-family G (WHITE), members 5 and 8 (*Abcg5* and *Abcg8*), which are involved in controlling sterol excretion from the liver [8], were down-regulated.

Genes involved in oxygen transport, namely hemoglobin alpha, adult chain 2 (*Hba-a2*) and hemoglobin, beta (*Hbb*), were down-regulated. Although no changes in blood hemoglobin levels were observed on day 3 of feeding the experimental diet, hepatic iron deficiency clearly influenced hemoglobin gene expression.

### Iron metabolism

The gene encoding hepcidin, a peptide hormone produced and released by the liver and the master regulator of iron homeostasis in the body by inhibiting the intestinal absorption of iron [9]–[12], was down-regulated. The gene encoding the transferrin receptor, a regulator of iron uptake into the cell [13], was not significantly changed.

### In situ hybridization analysis of G6pc and Hamp in the rat liver

**Figure 5:**
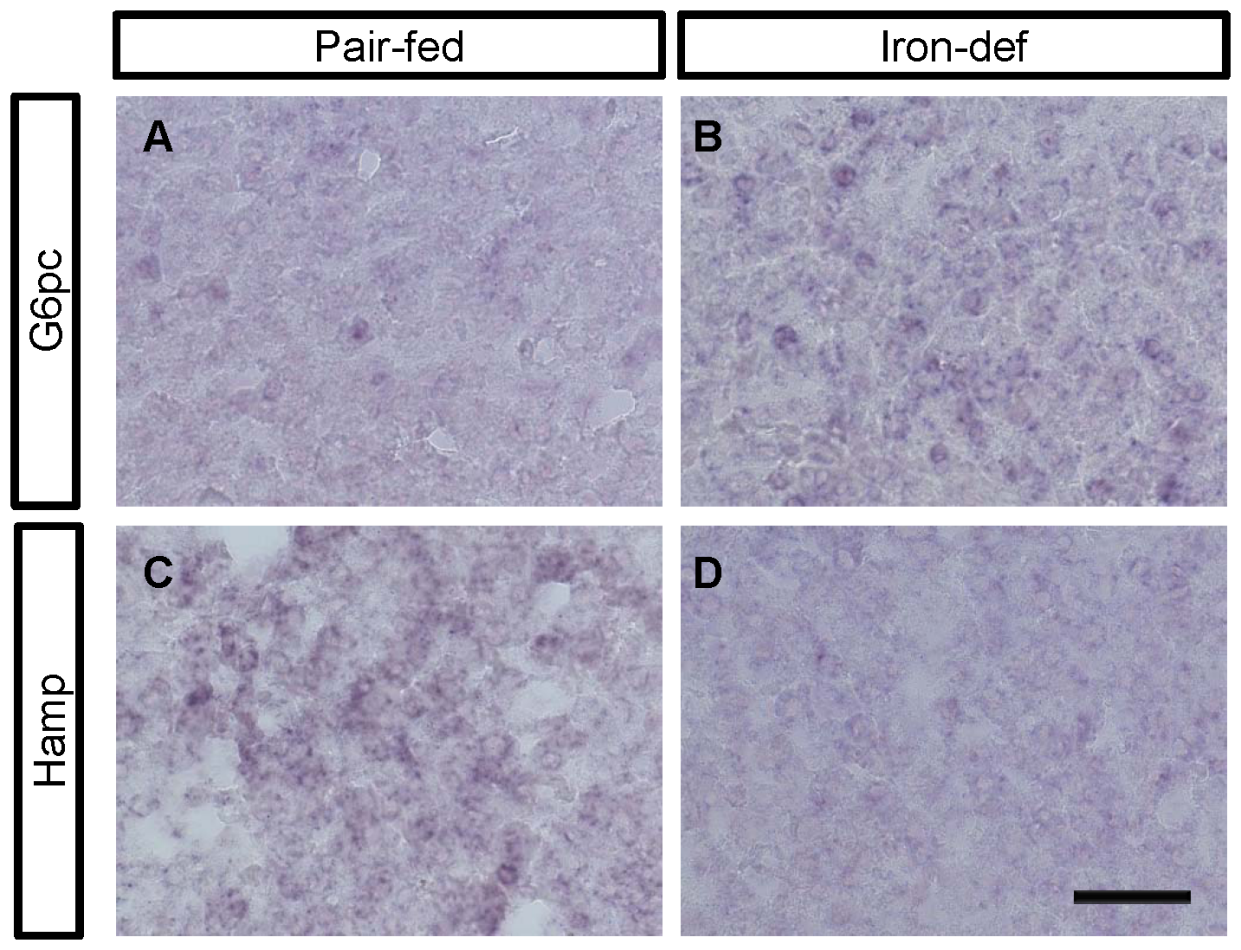
Expression of *G6pc* and *Hamp* in the livers of pair-fed and iron-deficient rats. Liver sections taken from pair-fed (A, C) and iron-deficient (B, D) rats were analyzed by *in situ* hybridization using *G6pc* (A, B) and *Hamp* (C, D) probes. The data are representative of experiments performed using three rats from each group. Scale bar = 50 µm.

*In situ* hybridization was performed to confirm the differential expression of *G6pc*, the rate-limiting enzyme in gluconeogenesis, and *Hamp*, an inhibitor of the intestinal uptake of iron, mRNAs in the rat liver in response to dietary iron-deficiency (Fig. 5). Weak *G6pc* signals (Fig. 5A) were observed in the rat liver in the pair-fed group. In contrast, clear *G6pc* signals (Fig. 5B) were observed in the iron-deficient diet group. Strong *Hamp* signals (Fig. 5C) were observed in the rat liver of the pair-fed group, and weak *Hamp* signals (Fig. 5D) existed in the iron-deficient diet group. These data were consistent with the DNA microarray data. No signals were detected in the negative control samples (data not shown).

### Comparison analysis

**Figure 6:**
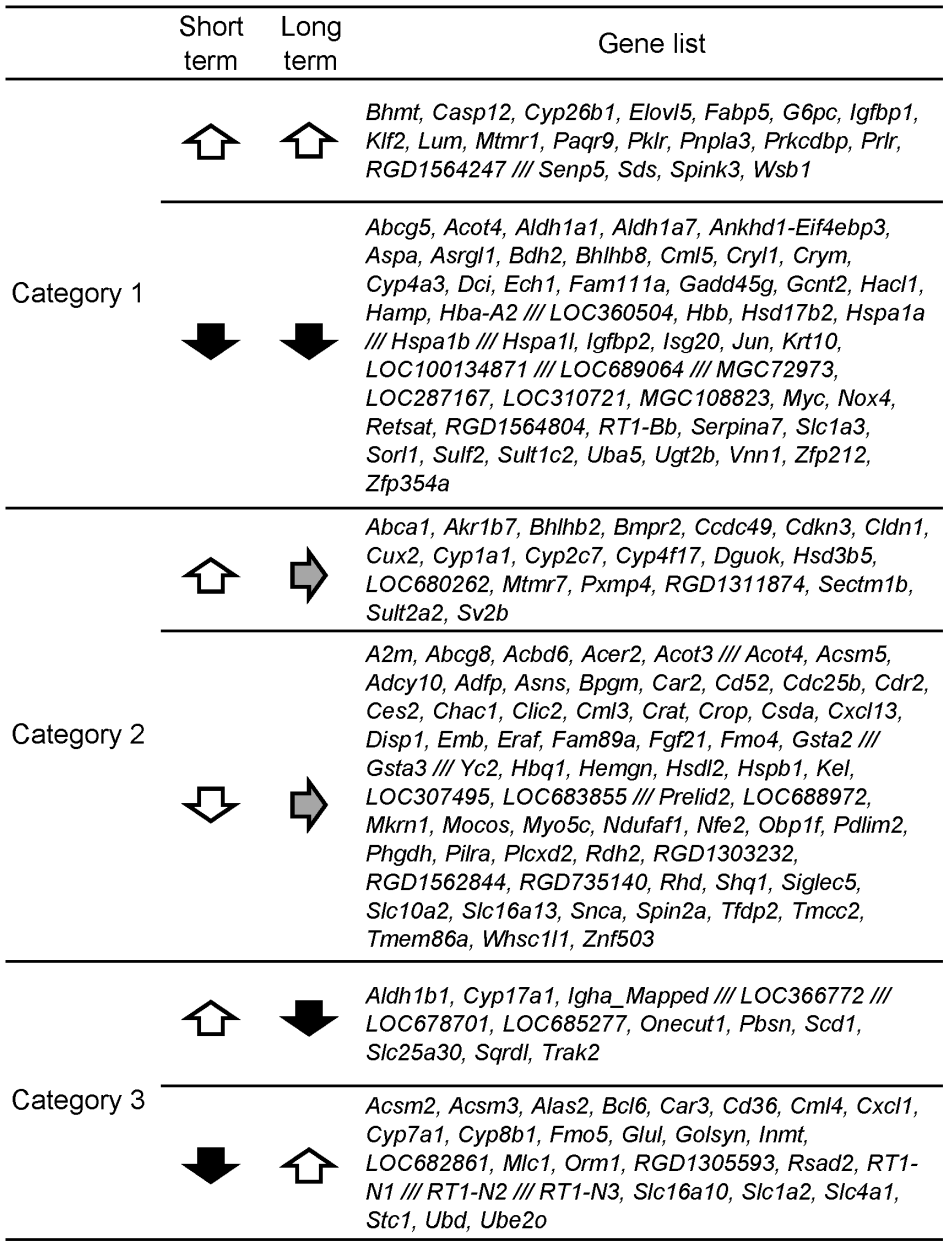
List of genes with expression changes in livers of rats fed an iron-deficient diet. White arrows: up-regulated gene expression, black arrows: down-regulated gene expression, gray arrows: no change.

The genes that were significantly differentially expressed following short-term feeding with an iron-deficient diet were compared to those from a previously described long-term experiment [3]. A gene list of short-term iron deficiency compared with long-term deficiency is provided in Figure 6. In category 1, the following genes were differentially regulated in response to both short- and long-term iron deficiency: up-relulation of *G6pc* and *Sds*, genes related to gluconeogenesis, and down-regulation of genes related to fatty acid oxidation, including *Hacl1*, *Bdh2*, *Dci*, *Ech* and *Cyp4a3*; the fatty acid biosynthesis gene *Acot4*; and the oxygen transport genes *Hba-a2* and *Hbb*. The acceleration of the early stages of gluconeogenesis led to increased serum glucose levels in the long-term iron-deficient rats [3].

In category 2, the genes encoding nuclear factor, erythroid-derived 2 (*Nfe2*); hemoglobin, theta 1 (*Hbq1*); erythroid associated factor (*Eraf*); and 2,3-bisphosphoglycerate mutase (*Bpgm*) were down-regulated only in the case of short-term iron deficiency.

In category 3, *Scd1*, an enzyme involved in fatty acid desaturation, was up-regulated, and enzymes involved in fatty acid catabolism, including *Acsm2*, *Acsm3* and *Cd36*, and cholesterol catabolism, including *Cyp7a1* and *Cyp8b1*, were down-regulated.

## Discussion

**Figure 7:**
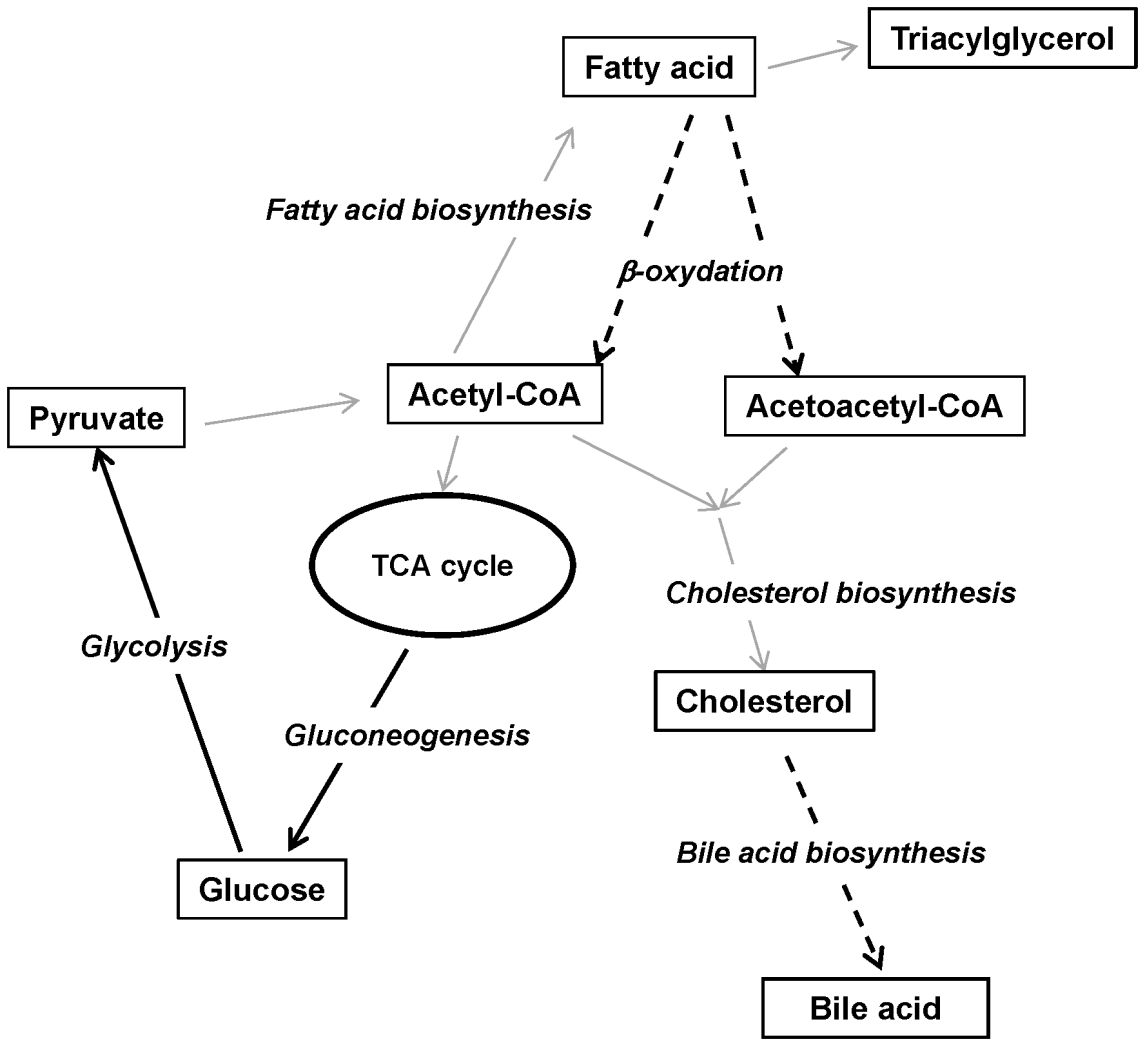
Potential hepatic metabolic changes due to an iron-deficient diet. Thick arrows: up-regulated gene expression. Broken arrows: down-regulated gene expression.

A dietary iron deficiency maintained for 3 days led to decreased iron and ferritin serum levels and increased TIBC but unaltered blood hemoglobin (Table 1). These results indicate that the rats experienced a certain degree of iron deficiency even though a state of anemia was not induced. In Japan, a major factor in the development of iron deficiency is unsatisfactory dietary iron intake [4]. Rats represent a useful experimental model for studying diet-induced non-anemic iron deficiency. In non-anemic iron deficiency, we observed changes in the expression of a variety of genes involved in metabolic processes (Fig. 7). Because iron is known to be a component of electron transport, the activity of the electron transport system is expected to be reduced in an iron-deficient state. This reduced activity may lead to the down-regulation of the TCA cycle. In this state, the activation of the Cori cycle is induced, leading to gluconeogenesis in liver. Consistent with this hypothesis, the expression of genes involved in gluconeogenesis was up-regulated in the livers of rats subjected to short-term iron deficiency. Moreover, because serum glucose levels increase in cases of anemia, iron deficiency likely induces gluconeogenesis rather than glycolysis [3]. The down-regulation of the TCA cycle should result in the down-regulation of lipid catabolism. In fact, fatty acid catabolism is reduced during short-term iron deficiency.

Some genes were common to both the short- and long-term iron deficiency models (category 1), some were specific to the short-term model (category 2), and the others were up-regulated in one model and down-regulated in the other (category 3) (Fig. 6). Collins et al. [14] showed that some novel genes were induced by iron deficiency during postnatal development in rats (from the suckling period to the adulthood intake stage). The authors identified genes that consistently displayed increased or decreased expression in all five different ages of iron-deficient rats. However, our study is the first to define the relationship between gene expression patterns and the degree of iron deficiency. Factors involved in iron absorption and oxygen transport were affected by both short- and long-term iron deficiencies. In particular, the gene expression of hepcidin, an inhibitor of intestinal iron absorption, was most significantly down-regulated, indicating an immediate increase in intestinal iron absorption. The genes that were differentially regulated specifically during short-term iron deficiency are involved in regulating oxygen affinity and hemoglobin at the transcriptional and post-translational levels. BPGM is known to possess mutase activity that enables the conversion of the glycolytic intermediate 1,3-bisphosphoglycerate (1,3-BPG) to 2,3-bisphosphoglycerate (2,3-BPG). 2,3-BPG decreases hemoglobin's affinity for oxygen [15], [16]. The down-regulation of *Bpgm* may therefore contribute to an increase in hemoglobin's oxygen affinity, thereby enhancing the efficiency of oxygen transport. The down-regulation of *Bpgm* may compensate for the decrease in hemoglobin. NFE2 is a transcription factor that regulates the expression of alpha- and beta-globin [17]. The down-regulation of *Nfe2* should result in the down-regulation of *Hba-a2* and *Hbb*. The gene expression of the other transcription factors that regulate globin genes, such as *Gata1* [18], was unaltered. ERAF, which is also known as alpha hemoglobin stabilizing protein (AHSP), enhances the subunit stability of alpha-hemoglobin and diminishes its participation in harmful redox reactions [19]. The down-regulation of *Eraf* may lead to an increased unfolding of alpha-hemoglobin proteins and it may subsequently inhibit the production of normal hemoglobin protein at the early stages. *Eraf* expression was differentially regulated at different iron levels. Iron is known to destabilize endogenous AHSP mRNA in K562 human erythroleukemia cells, most likely by causing the release of iron regulatory proteins (IRPs) from an iron responsive element (IRE)-like structure in the 3′ UTR. Iron also simultaneously increased the levels of nascent primary transcripts [20]. These two mechanisms may explain the temporary down-regulation of *Eraf* expression. The changes in gene expression patterns in category 3 are attributed to homeostatic compensation.

In this study, we found that rats fed an iron-deficient diet for only 3 days reached a state of non-anemic iron deficiency. The gene expression analysis indicated that decreased liver iron storage dramatically influenced hepatic gene expression patterns. It will be important to examine the influence of different iron storage levels, induced by either depletion or excess iron intake, on hepatic gene expression patterns. A complete understanding of how iron deficiency affects physiological processes requires further investigation.

## Materials and Methods

### Animals and diets

Three-week-old male Sprague-Dawley rats were purchased from Charles River Japan (Kanagawa, Japan) and housed in a room maintained at 24±1°C and 40±5% humidity with a 12-h light/dark cycle (light 08:00–20:00; dark 20:00–08:00). The rats were given a normal diet (Research Diets, Inc., New Brunswick, NJ, USA) (Table 3) and deionized distilled water ad libitum prior to the beginning of the study. The normal diet, 48 ppm iron, was based on the AIN-93G diet; Avicel was used in place of cellulose, which may contain trace amounts of iron. On day 3 of the acclimatization period, the food was removed at 17:00, and normal feeding was conducted between 09:00 and 17:00 for the next 4 days to synchronize the feeding behaviors; this feeding was continued until one day before sacrifice. On day 8, the rats were divided into two groups with similar average body weights. The rats in the iron-deficient group (n = 5) were given ad libitum access to an iron-deficient diet, which contained approximately 3 ppm iron, the food was prepared by removing the iron (ferric citrate) from the normal diet (Table 3). The rats in the pair-fed group (n = 5) were fed the normal diet by pair feeding. On days 8 and 10 (i.e., on days 1 and 3 of feeding the experimental diet), hemoglobin levels were measured in blood samples that were collected from the tail vein of each rat. On day 3 of feeding the experimental diet, the rats were sacrificed under anesthesia 1.5 h after feeding, and the livers were excised and immediately immersed in RNAlater (Applied Biosystems Japan, Tokyo, Japan). Blood was obtained from the carotid artery. All of the animal experimental protocols were approved by the Animal Use Committee of the Faculty of Agriculture at the University of Tokyo (approval number: P09-283).

**Table 3:** Diet composition.

	Normal diet (g/kg)	Iron-deficient diet (g/kg)
Casein	200.000	200.000
L-cystine	3.000	3.000
Corn starch	397.486	397.486
Maltodextrin 10	132.000	132.000
Sucrose	100.000	100.000
Avicel, PH101^1^	50.000	50.000
Soybean oil	70.000	70.000
τ-Butylhydroquinone	0.014	0.014
Mineral mix S18706^2^	35.000	35.000
Ferric citrate	0.212	0.000
Vitamin mix V10037^3^	10.000	10.000
Choline bitartrate	2.500	2.500
Total	1000.212	1000.000

1 Avicel PH101: sulfite cellulose.

2 Mineral mix S18706 formulated according to AIN-93 without ferric citrate.

3 Vitamin mix V10037 formulated according to AIN-93.

### Measurement of biochemical parameters for blood, serum and liver

Hemoglobin levels were measured using the Wako Hemoglobin B test (Wako Pure Chemical Industries, Osaka, Japan). Serum ferritin levels were measured using the Rat Ferritin EIA kit (Mitsubishi Chemical Medience Corporation, Tokyo, Japan). Total iron binding capacity (TIBC), iron, total cholesterol, triacylglycerol, glucose and pyruvate levels were measured from serum with the help of Nagahama Life Science (Shiga, Japan), and hepatic iron, cholesterol, bile acid and triacylglycerol were measured with the help of the Material Research Center (Chiba, Japan). The statistical significance of between-group differences was assessed with Student's *t-*test; the differences were considered to be significant if *P*<0.05 and highly significant if *P*<0.01.

### Isolation of total RNA

Total RNA was isolated from each liver sample using the TRIzol reagent (Invitrogen Japan K.K., Tokyo, Japan) and then purified using an RNeasy Mini Kit (Qiagen K.K., Tokyo, Japan). The quality and quantity of the total RNA were spectrophotometrically evaluated with an Agilent 2100 Bioanalyzer (Agilent Technologies Japan, Tokyo, Japan) using an RNA 6000 Nano Series II Kit (Agilent Technologies Japan, Tokyo, Japan). The RNA integrity number (RIN) was computed using the 2100 Expert software program (Agilent Technologies Japan, Tokyo, Japan) to indicate the integrity of the total RNA samples on a scale of 1–10 [21]; the RIN of the total RNA isolated from each liver was greater than 8.5.

### DNA microarray assay

The rat total RNA samples were processed for DNA microarray analysis as described previously [3]. In brief, cDNA was synthesized from 2 µg of purified total RNA, and biotinylated cRNA was then transcribed using T7 RNA polymerase by using GeneChip® Expression 3′ Amplification One-Cycle Target Labeling and Control Reagents (Affymetrix, Santa Clara, CA, USA). The cRNA quality was evaluated with an Agilent 2100 Bioanalyzer. The cRNA was fragmented and hybridized to a GeneChip® Rat Genome 230 2.0 Array (Affymetrix, Santa Clara, CA, USA) that included more than 31,000 probe sets. Following hybridization at 45°C for 16 h, the array was washed and stained with phycoerythrin using the GeneChip® Fluidics Station 450 (Affymetrix, Santa Clara, CA, USA). Fluorescence signals were scanned using a GeneChip® Scanner 3000 7G (Affymetrix, Santa Clara, CA, USA). The Affymetrix® GeneChip® Command Console® (AGCC) software program (Affymetrix, Santa Clara, CA, USA) was used to convert the array images to the intensity values for each probe (CEL files). All of the microarray data are MIAME compliant and have been deposited in a MIAME compliant database, the National Center for Biotechnology Information (NCBI) Gene Expression Omnibus (http://www.ncbi.nlm.nih.gov/geo/, GEO Series accession number GSE30533), as detailed on the MGED Society website (http://www.mged.org/Workgroups/MIAME/miame.html).

### DNA microarray data analysis

The CEL files were quantified by calculating a robust multiarray average (RMA) [22] using the statistical language R [23], version 2.7.1, and Bioconductor [24], version 2.2. Hierarchical clustering was then performed using the pvclust() function [25] in R. To identify the probe sets that were differentially expressed between the groups, the rank products (RP) method [26] was applied to the RMA-quantified data. A recent study reported that the RP method in conjunction with an RMA preprocessing algorithm is one of the best combinations to accurately detect differentially expressed probe sets [27]. Probe sets with a false discovery rate (FDR)<0.05 were considered to be differentially expressed. The annotation file for the Rat Genome 230 2.0 Array was downloaded from the Affymetrix website (November 15, 2009, Rat230_2.na30.annot.csv).

### Gene ontology analysis

The selected probe sets were functionally classified according to the Biological Process in Gene Ontology (GO) using the Functional Annotation Tool of the Database for Annotation, Visualization, and Integrated Discovery (DAVID) [28]. The probe set IDs provided by Affymetrix were used as the input data format. In the probe set list manager on the DAVID website (http://david.abcc.ncifcrf.gov/), we selected the species option to limit the annotations exclusively to *RATTUS NORVEGICUS*. For the population manager option, the Rat Expression Array 230 2.0 platform was selected as the background. The Functional Annotation Chart was analyzed on the basis of Biological Process in GO, GOTERM_BP_ALL. To extract the statistically overrepresented GO terms in differentially expressed genes, we used EASE scores, which are modified Fisher's exact test *P*-values [29]. The Benjamini and Hochberg false discovery rate (FDR) [30] was used to correct for multiple tests. A GO term with an FDR-corrected *P*-value<0.05 was considered to be significantly enriched. We used the online analysis application QuickGO (https://www.ebi.ac.uk/QuickGO/) [31] to determine the hierarchical structure of the selected GO terms.

### In situ hybridization

Liver tissue from pair-fed and iron-deficient rats was embedded in Tissue-Tek O. C. T. compound (Sakura Finetechnical, Tokyo, Japan) and frozen in liquid nitrogen immediately after excision. Frozen tissue samples were each sectioned into 14 µm-thick slices. The sections were fixed with 4% paraformaldehyde (PFA) and treated with 1–3% hydrogen peroxide. PFA-fixed sections were treated with Histo VT One (Nacalai Tesque, Kyoto, Japan) for 20 min and acetylated in 0.1 M triethanolamine containing 0.25% acetic anhydride for 10 min. The sections were prehybridized with salmon sperm DNA for 2 h at 65°C and hybridized with antisense riboprobes in hybridization buffer (50% formamide, 5× SSC, 5× Denhardt's solution, 500 µg/ml salmon sperm DNA, 250 µg/ml t-RNA and 1 mM DTT) overnight at 65°C. After hybridization, the sections were washed in 5× SSC at 65°C, 0.2× SSC at 65°C and blocked in blocking solution containing 0.5% blocking reagent (GE Healthcare Japan, Tokyo, Japan). For labeling, the signals were developed using an alkaline phosphatase-conjugated anti-digoxigenin antibody (1∶1000, Roche Applied Science, IN, USA) and nitro blue tetrazolium chloride/5-bromo-4-chloro-3-indolyl phosphate as chromogenic substrates. Stained images were obtained with a Leica DM RA2 (Leica Microsystems, Wetzlar, Germany) microscope equipped with a Leica DFC290HD (Leica Microsystems, Wetzlar, Germany). The antisense riboprobe for *Hamp* was synthesized from the fragments subcloned into the pBluescript II SK-(-) vector (Stratagene, LA Jolla, CA, USA), and *G6pc* was derived from the cDNA clone Cat. MRN1768-98078553, which was purchased from Thermo Scientific (CO, USA).

### Comparison with long-term iron deficiency

The genes that were significantly differentially expressed after short-term feeding with an iron-deficient diet were compared with those from a previously described long-term experiment [3]. The changes in gene expression were divided into the following three categories: category 1, which consisted of genes common to both cases; category 2, which consisted of genes specific to the short-term case; and category 3, which consisted of genes with opposite profiles in the short- and long-term cases.

## Supporting Information

Figure S1 **Hierarchical clustering dendrograms from the RMA-quantified DNA microarray data.** Pair-fed, pair-fed group; Iron-def, iron-deficient group. The numbers represent independent samples. The vertical scale represents between-cluster distances.
(TIFF)

Table S1 **List of all differentially expressed probe sets in iron-deficient rats.**
(DOCX)
