# Supplementary material for: Influence of a Short-Term Iron-Deficient Diet on Hepatic Gene Expression Profiles in Rats
Source: PLoS One. 2013 Jun 5;8(6):e65732. doi: 10.1371/journal.pone.0065732 (PMC3674005; doi:10.1371/journal.pone.0065732)
Supplement: Table S1 — List of all differentially expressed probe sets in iron-deficient rats. (DOCX) [file pone.0065732.s002.docx]

**Table S1** List of all differentially expressed probe sets in iron-deficient rats

| Probe set ID | Gene Symbol | Change |
| --- | --- | --- |
| 1394490_at | Abca1 | Up |
| 1368569_at | Akr1b7 | Up |
| 1383472_at | Aldh1b1 | Up |
| 1369415_at | Bhlhb2 | Up |
| 1387665_at | Bhmt | Up |
| 1376843_at | Bmpr2 | Up |
| 1387605_at | Casp12 | Up |
| 1374888_at | Ccdc49 | Up |
| 1372685_at | Cdkn3 | Up |
| 1396150_at | Cldn1 | Up |
| 1382202_at | Cux2 | Up |
| 1387123_at | Cyp17a1 | Up |
| 1370269_at | Cyp1a1 | Up |
| 1384392_at | Cyp26b1 | Up |
| 1370241_at | Cyp2c7 | Up |
| 1392720_at | Cyp4f17 | Up |
| 1379435_at | Dguok | Up |
| 1387630_at | Elovl5 | Up |
| 1370281_at | Fabp5 | Up |
| 1370725_a_at | G6pc | Up |
| 1386944_a_at | G6pc | Up |
| 1369465_at | Hsd3b5 | Up |
| 1368160_at | Igfbp1 | Up |
| 1374334_at | Igha_mapped /// LOC366772 /// LOC678701 | Up |
| 1386041_a_at | Klf2 | Up |
| 1376569_at | Klf2 | Up |
| 1375988_at | LOC680262 | Up |
| 1384035_at | LOC685277 | Up |
| 1367749_at | Lum | Up |
| 1391512_at | Mtmr1 | Up |
| 1374883_at | Mtmr7 | Up |
| 1387760_a_at | Onecut1 | Up |
| 1382569_at | Paqr9 | Up |
| 1387191_at | Pbsn | Up |
| 1368651_at | Pklr | Up |
| 1380013_at | Pnpla3 | Up |
| 1367691_at | Prkcdbp | Up |
| 1370384_a_at | Prlr | Up |
| 1370789_a_at | Prlr | Up |
| 1369493_at | Prlr | Up |
| 1383117_at | Pxmp4 | Up |
| 1372387_at | RGD1311874 | Up |
| 1391262_at | RGD1564247 /// Senp5 | Up |
| 1370355_at | Scd1 | Up |
| 1369864_a_at | Sds | Up |
| 1376976_at | Sectm1b | Up |
| 1381973_at | Slc25a30 | Up |
| 1390416_at | Slc25a30 | Up |
| 1387193_a_at | Spink3 | Up |
| 1368447_x_at | Spink3 | Up |
| 1389142_at | Sqrdl | Up |
| 1387936_at | Sult2a2 | Up |
| 1369628_at | Sv2b | Up |
| 1370176_at | Trak2 | Up |
| 1374204_at | Wsb1 | Up |
| 1371298_at | --- | Up |
| 1372700_at | --- | Up |
| 1372727_at | --- | Up |
| 1373628_at | --- | Up |
| 1374276_at | --- | Up |
| 1375647_at | --- | Up |
| 1376267_at | --- | Up |
| 1376944_at | --- | Up |
| 1377650_at | --- | Up |
| 1378960_at | --- | Up |
| 1379859_at | --- | Up |
| 1379888_at | --- | Up |
| 1380115_at | --- | Up |
| 1381187_at | --- | Up |
| 1381264_at | --- | Up |
| 1382431_at | --- | Up |
| 1382944_at | --- | Up |
| 1385029_at | --- | Up |
| 1385355_at | --- | Up |
| 1388348_at | --- | Up |
| 1390117_at | --- | Up |
| 1390622_at | --- | Up |
| 1391166_at | --- | Up |
| 1391544_at | --- | Up |
| 1391718_at | --- | Up |
| 1392612_at | --- | Up |
| 1392702_at | --- | Up |
| 1392767_at | --- | Up |
| 1393302_at | --- | Up |
| 1393759_at | --- | Up |
| 1396155_at | --- | Up |
| 1397153_at | --- | Up |
| 1397222_at | --- | Up |
| 1397249_at | --- | Up |
| 1397855_at | --- | Up |
| 1398566_at | --- | Up |
| 1367794_at | A2m | Down |
| 1369455_at | Abcg5 | Down |
| 1369440_at | Abcg8 | Down |
| 1374555_at | Acbd6 | Down |
| 1379846_at | Acer2 | Down |
| 1378169_at | Acot3 /// Acot4 | Down |
| 1377037_at | Acot4 | Down |
| 1370436_at | Acsm2 | Down |
| 1383303_at | Acsm3 | Down |
| 1377407_at | Acsm5 | Down |
| 1369337_at | Adcy10 | Down |
| 1390850_at | Adfp | Down |
| 1367985_at | Alas2 | Down |
| 1387022_at | Aldh1a1 | Down |
| 1368718_at | Aldh1a7 | Down |
| 1373692_at | Ankhd1-Eif4ebp3 | Down |
| 1387925_at | Asns | Down |
| 1368563_at | Aspa | Down |
| 1387966_at | Asrgl1 | Down |
| 1379368_at | Bcl6 | Down |
| 1372613_at | Bdh2 | Down |
| 1387212_at | Bhlhb8 | Down |
| 1383551_at | Bpgm | Down |
| 1388544_at | Bpgm | Down |
| 1386922_at | Car2 | Down |
| 1394551_at | Car3 | Down |
| 1367689_a_at | Cd36 | Down |
| 1368338_at | Cd52 | Down |
| 1370034_at | Cdc25b | Down |
| 1374139_at | Cdr2 | Down |
| 1368905_at | Ces2 | Down |
| 1389573_at | Chac1 | Down |
| 1381993_at | Clic2 | Down |
| 1370991_at | Cml3 | Down |
| 1387336_at | Cml4 | Down |
| 1388176_at | Cml5 | Down |
| 1371886_at | Crat | Down |
| 1377694_at | Crop | Down |
| 1376051_at | Cryl1 | Down |
| 1368059_at | Crym | Down |
| 1370376_a_at | Csda | Down |
| 1387316_at | Cxcl1 | Down |
| 1398390_at | Cxcl13 | Down |
| 1370397_at | Cyp4a3 | Down |
| 1368458_at | Cyp7a1 | Down |
| 1368435_at | Cyp8b1 | Down |
| 1367659_s_at | Dci | Down |
| 1374219_at | Disp1 | Down |
| 1386885_at | Ech1 | Down |
| 1368541_at | Emb | Down |
| 1389160_at | Eraf | Down |
| 1371970_at | Fam111a | Down |
| 1377307_at | Fam89a | Down |
| 1387643_at | Fgf21 | Down |
| 1395026_at | Fmo4 | Down |
| 1383248_at | Fmo5 | Down |
| 1388792_at | Gadd45g | Down |
| 1374903_at | Gcnt2 | Down |
| 1367633_at | Glul | Down |
| 1386870_at | Glul | Down |
| 1393510_at | Golsyn | Down |
| 1371089_at | Gsta2 /// Gsta3 /// Yc2 | Down |
| 1371012_at | Hacl1 | Down |
| 1387396_at | Hamp | Down |
| 1388608_x_at | Hba-a2 /// LOC360504 | Down |
| 1370240_x_at | Hba-a2 /// LOC360504 | Down |
| 1367553_x_at | Hbb | Down |
| 1390991_at | Hbq1 | Down |
| 1387756_s_at | Hemgn | Down |
| 1380824_at | Hook3 | Down |
| 1387156_at | Hsd17b2 | Down |
| 1397468_at | Hsdl2 | Down |
| 1368247_at | Hspa1a /// Hspa1b /// Hspa1l | Down |
| 1367577_at | Hspb1 | Down |
| 1367648_at | Igfbp2 | Down |
| 1373975_at | Inmt | Down |
| 1390507_at | Isg20 | Down |
| 1389528_s_at | Jun | Down |
| 1369788_s_at | Jun | Down |
| 1374330_at | Kel | Down |
| 1373254_at | Krt10 | Down |
| 1371245_a_at | LOC100134871 /// LOC689064 /// MGC72973 | Down |
| 1371102_x_at | LOC100134871 /// LOC689064 /// MGC72973 | Down |
| 1375519_at | LOC287167 | Down |
| 1383888_at | LOC307495 | Down |
| 1382907_at | LOC310721 | Down |
| 1385659_at | LOC682861 | Down |
| 1383692_at | LOC683855 /// Prelid2 | Down |
| 1375842_at | LOC688972 | Down |
| 1373992_at | MGC108823 | Down |
| 1373496_at | Mkrn1 | Down |
| 1379325_at | Mkrn1 | Down |
| 1376702_at | Mlc1 | Down |
| 1372683_at | Mocos | Down |
| 1368308_at | Myc | Down |
| 1391018_at | Myo5c | Down |
| 1393689_at | Ndufaf1 | Down |
| 1375040_at | Nfe2 | Down |
| 1368543_at | Nox4 | Down |
| 1388014_at | Obp1f | Down |
| 1368731_at | Orm1 | Down |
| 1375367_at | Pdlim2 | Down |
| 1367811_at | Phgdh | Down |
| 1398589_at | Pilra | Down |
| 1381206_at | Plcxd2 | Down |
| 1370566_at | Rdh2 | Down |
| 1370806_at | Retsat | Down |
| 1378802_at | RGD1303232 | Down |
| 1388306_at | RGD1305593 | Down |
| 1376958_at | RGD1562844 | Down |
| 1392485_at | RGD1564804 | Down |
| 1394327_at | RGD1564804 | Down |
| 1393606_at | RGD735140 | Down |
| 1370079_at | Rhd | Down |
| 1370913_at | Rsad2 | Down |
| 1371033_at | RT1-Bb | Down |
| 1387839_at | RT1-N1 /// RT1-N2 /// RT1-N3 | Down |
| 1371143_at | Serpina7 | Down |
| 1378940_at | Shq1 | Down |
| 1385465_at | Siglec5 | Down |
| 1368745_at | Slc10a2 | Down |
| 1370548_at | Slc16a10 | Down |
| 1382003_at | Slc16a13 | Down |
| 1369693_a_at | Slc1a2 | Down |
| 1368565_at | Slc1a3 | Down |
| 1387656_at | Slc4a1 | Down |
| 1367977_at | Snca | Down |
| 1393933_at | Sorl1 | Down |
| 1377457_a_at | Sorl1 | Down |
| 1384023_at | Spin2a | Down |
| 1396101_at | Stc1 | Down |
| 1388753_at | Sulf2 | Down |
| 1377672_at | Sult1c2 | Down |
| 1369531_at | Sult1c2 | Down |
| 1392405_at | Tfdp2 | Down |
| 1374160_at | Tmcc2 | Down |
| 1373309_at | Tmem86a | Down |
| 1372120_at | Uba5 | Down |
| 1368762_at | Ubd | Down |
| 1373438_at | Ube2o | Down |
| 1385247_at | Ugt2b | Down |
| 1389253_at | Vnn1 | Down |
| 1377897_at | Whsc1l1 | Down |
| 1380094_a_at | Zfp212 | Down |
| 1368877_at | Zfp354a | Down |
| 1393990_at | Znf503 | Down |
| 1371391_at | --- | Down |
| 1376071_at | --- | Down |
| 1376153_at | --- | Down |
| 1376226_at | --- | Down |
| 1376637_at | --- | Down |
| 1376829_at | --- | Down |
| 1377451_at | --- | Down |
| 1378052_at | --- | Down |
| 1378472_at | --- | Down |
| 1378800_at | --- | Down |
| 1379363_at | --- | Down |
| 1380529_at | --- | Down |
| 1381083_at | --- | Down |
| 1382173_at | --- | Down |
| 1382335_at | --- | Down |
| 1382644_at | --- | Down |
| 1382703_at | --- | Down |
| 1384574_at | --- | Down |
| 1385205_at | --- | Down |
| 1386695_at | --- | Down |
| 1388146_at | --- | Down |
| 1389486_at | --- | Down |
| 1390457_at | --- | Down |
| 1390458_at | --- | Down |
| 1390735_at | --- | Down |
| 1391202_at | --- | Down |
| 1391654_at | --- | Down |
| 1391912_at | --- | Down |
| 1392246_at | --- | Down |
| 1392637_at | --- | Down |
| 1392788_at | --- | Down |
| 1393108_at | --- | Down |
| 1393455_at | --- | Down |
| 1393508_at | --- | Down |
| 1393559_at | --- | Down |
| 1394101_at | --- | Down |
| 1395324_at | --- | Down |
| 1395546_at | --- | Down |
| 1396420_at | --- | Down |
| 1398560_at | --- | Down |
